# Expression profiling of genes modulated by minocycline in a rat model of neuropathic pain

**DOI:** 10.1186/1744-8069-10-47

**Published:** 2014-07-19

**Authors:** Ewelina Rojewska, Michal Korostynski, Ryszard Przewlocki, Barbara Przewlocka, Joanna Mika

**Affiliations:** 1 Department of Pain Pharmacology, Institute of Pharmacology, Polish Academy of Sciences, Krakow, Poland; 2 Department of Molecular Neuropharmacology, Institute of Pharmacology, Polish Academy of Sciences, Krakow, Poland

**Keywords:** Microarray DNA, Neuropathic pain, Minocycline, Pain-related genes

## Abstract

**Background:**

The molecular mechanisms underlying neuropathic pain are constantly being studied to create new opportunities to prevent or alleviate neuropathic pain. The aim of our study was to determine the gene expression changes induced by sciatic nerve chronic constriction injury (CCI) that are modulated by minocycline, which can effectively diminish neuropathic pain in animal studies. The genes associated with minocycline efficacy in neuropathic pain should provide insight into the etiology of neuropathic pain and identify novel therapeutic targets.

**Results:**

We screened the ipsilateral dorsal part of the lumbar spinal cord of the rat CCI model for differentially expressed genes. Out of 22,500 studied transcripts, the abundance levels of 93 transcripts were altered following sciatic nerve ligation. Percentage analysis revealed that 54 transcripts were not affected by the repeated administration of minocycline (30 mg/kg, *i.p*.), but the levels of 39 transcripts were modulated following minocycline treatment. We then selected two gene expression patterns, B1 and B2. The first transcription pattern, B1, consisted of 10 mRNA transcripts that increased in abundance after injury, and minocycline treatment reversed or inhibited the effect of the injury; the B2 transcription pattern consisted of 7 mRNA transcripts whose abundance decreased following sciatic nerve ligation, and minocycline treatment reversed the effect of the injury. Based on the literature, we selected seven genes for further analysis: *Cd40*, *Clec7a*, *Apobec3b*, *Slc7a7*, and *Fam22f* from pattern B1 and *Rwdd3* and *Gimap5* from pattern B2. Additionally, these genes were analyzed using quantitative PCR to determine the transcriptional changes strongly related to the development of neuropathic pain; the ipsilateral DRGs (L4-L6) were also collected and analyzed in these rats using qPCR.

**Conclusion:**

In this work, we confirmed gene expression alterations previously identified by microarray analysis in the spinal cord and analyzed the expression of selected genes in the DRG. Moreover, we reviewed the literature to illustrate the relevance of these findings for neuropathic pain development and therapy. Further studies are needed to elucidate the roles of the individual genes in neuropathic pain and to determine the therapeutic role of minocycline in the rat neuropathic pain model.

## Background

Studies conducted in recent years have shown that multiple endogenous factors initiate and regulate neuropathic pain, and neuroimmune interactions play important roles in this process, which may be one reason for the loss of efficacy for many analgesics [1-5]. Recent reports suggest that inhibitors of microglial activation and cytokine synthesis, including minocycline, pentoxyfylline and propentofylline, may significantly inhibit the development of neuropathic pain in animal models [6-10]. The effects of these substances are a result of the decreased secretion of numerous cytokines due to the reduced activation of the microglia or other cells [6,7,9,11]. Administration of these inhibitors also enhances the effect of analgesic drugs, such as morphine [7,12-14].

Our previous data highlighted the importance of immune response- and microglia activation-related genes in the development of neuropathic pain in the spinal dorsal horn and their involvement in the persistence of its symptoms [4,7,14,15]. Therefore, the aim of the present study was to determine which of the genes that are altered after sciatic nerve injury are modulated by minocycline. Minocycline is a strong modulator of the neuroimmune response and readily permeates the blood–brain barrier [12,16-21]. Minocycline is effective at reducing allodynia in animal models, and therefore, it appears to be a promising substance that could be used in analgesia, as confirmed by experimental studies [7,15,22]. This drug is also effective in the treatment of neurodegenerative diseases in a clinical setting [23]; however, it has not been used in the treatment of neuropathic pain. The neuroprotective properties of minocycline have been observed in spinal cord injury models [24], hypoxia models [25,26] and in Huntington’s and Parkinson’s diseases in the clinical setting [20,21,25,26]. Minocycline was shown to alleviate neuropathic pain symptoms by inhibiting the activation of the microglia within the spinal cord, leading to the reduced release of pro-inflammatory agents, which often exhibit pronociceptive properties [9,27]. Additionally, minocycline is believed to promote the maintenance of the blood–brain barrier integrity by reducing the expression of chemokine receptors and metalloproteinases, as well as by reducing the production of reactive oxygen species [28]. By affecting the degree of activation of the microglia, minocycline enhances the analgesic effects of morphine, which has been shown to be less effective in neuropathic pain models [7,12-14,29].

Microarray analysis for global gene expression indicated that both neuropathic and inflammatory pain are associated with a dramatic shift in the regulation of many genes in the spinal cord and dorsal root ganglia DRG [30,31].

Therefore, the aim of the present study was to determine which of the genes that were altered after sciatic nerve injury are modulated by minocycline. As DNA microarray experiments allowed us to identify numerous transcripts that are important for the development of neuropathy and are possible targets for drug therapy, we planned the experiments to study the molecular basis of the inhibitory effects of minocycline on the development of neuropathic pain. We performed behavioral studies and microarray screening for genes in the lumbar section of the rat spinal cord in the rat model of neuropathic pain (*chronic constriction injury to the sciatic nerve*, *CCI*), and we identified genes that are associated with minocycline efficacy in neuropathic pain. The changes in the abundance of some transcripts in the ipsilateral dorsal part of the lumbar spinal cord after sciatic nerve ligation and the modulatory role of minocycline were confirmed by quantitative PCR analysis of samples independent from those used for the microarray analysis, as well as DRG samples from the same group of rats subjected to CCI.

## Results

### The effect of minocycline *i.p*. administration on the development of mechanical allodynia and thermal hyperalgesia in CCI-exposed rats

**Figure 1:**
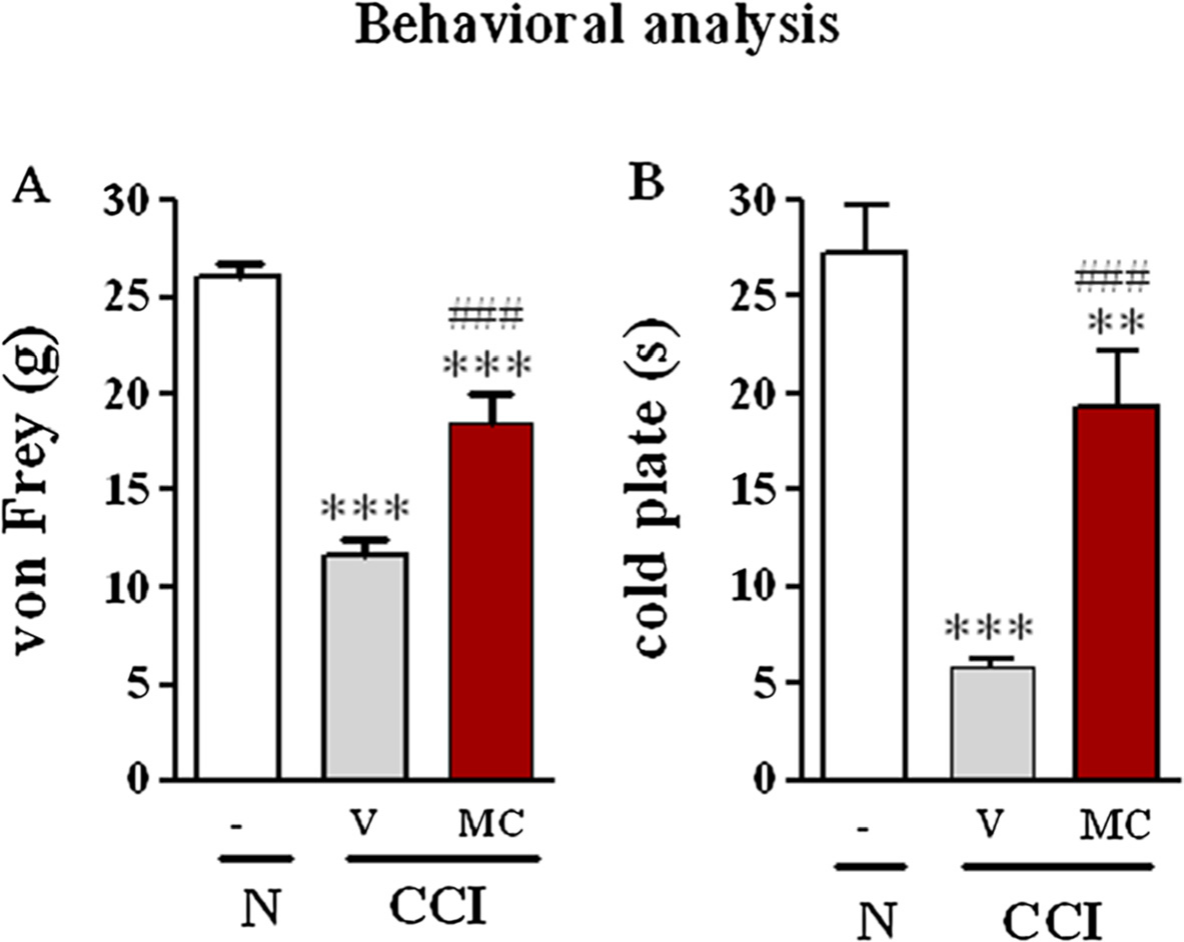
**The effect of minocycline on the development of allodynia and hyperalgesia 7 days after CCI.** Influence of preemptive and repeated administration of minocycline (30 mg/kg; *i.p*.; 16 h and 1 h before CCI and then for 7 days twice daily) on the development of allodynia (**A**; von Frey test) and hyperalgesia (**B**; cold plate test) seven days after CCI in rats. The data are presented as the mean ± S.E.M (10–12 rat per group). Allodynia and hyperalgesia were assessed 60 min after drug administration. The inter-group differences were analyzed using an ANOVA and Bonferroni’s multiple comparison test; **p < 0.01 and ***p < 0.001 indicate a significant difference when compared to the control (naïve rats), and ^###^p < 0.001 indicates a significant difference when compared to the V-CCI rats (ANOVA, Bonferroni’s test). Naive (N), vehicle (V; water for injection), minocycline (MC).

Unilateral, loose ligation of the sciatic nerve led to the development of symptoms typical of neuropathic pain, such as allodynia (Figure 1A) and hyperalgesia (Figure 1B). In the von Frey test, strong tactile allodynia on the paw ipsilateral to the injury was observed on day seven after CCI; at this time, the ipsilateral paw responded to a stimulation of 14.8 ± 0.7 g (Figure 1A), compared to the reactions of the hind paws of naive rats to 26.0 ± 0.7 g. The strongest hyperalgesia was observed on the seventh day in the cold plate test (Figure 1B). At this time, the ipsilateral paw reacted after 6.2 ± 0.7 s (Figure 1B), compared to reaction after 27.3 ± 2.4 s in the naive rats.

Administration of minocycline led to a significant reduction in the above described symptoms. Preemptive and repeated treatment with minocycline (twice daily; 30 mg/kg *i.p*.) significantly attenuated the allodynia to 18.4 ± 1.5 g (Figure 1A) and the hyperalgesia to 19.3 ± 2.9 s (Figure 1B) on day seven after CCI.

### Profiling the gene expression alterations following minocycline *i.p*. administration to CCI-exposed rats

**Figure 2:**
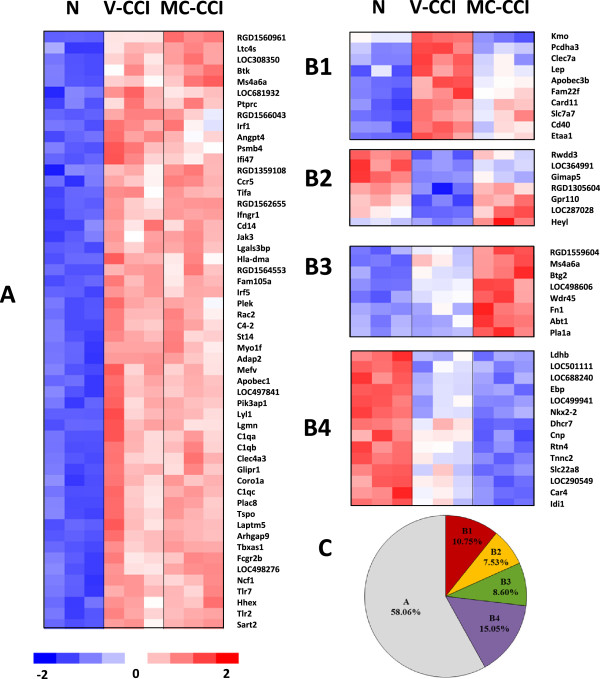
**Profiling the gene expression alterations following minocycline i.p. administration to CCI**-**exposed rats.** Gene expression changes 7 days after nerve injury and administration of minocycline (30 mg/kg; *i.p*.; 16 h and 1 h before CCI and then for 7 days twice daily). The statistical analysis was performed at an ANOVA threshold value of p < 0.001 (N *vs*. V-CCI *vs*. MC-CCI). The results of the cDNA microarray analysis are presented in the form of a heat map. The individual columns represent the respective microarrays, while the individual rows represent the regulated genes. The intensity of the color reflects the relative abundance of the transcript and is proportional to the standard deviation from the in-row average, as indicated in the legend below the heat map. **(A)** The gene pattern represents changes in the abundance levels of 54 mRNA transcripts induced in the lumbar spinal segment on day 7 of neuropathic pain; these abundance levels of these genes were not affected by the addition of minocycline (30 mg/kg; 16 h and 1 h before sciatic nerve damage and then twice daily for 7 days). **(B)** Gene pattern B represents changes in the abundance levels of 39 mRNA transcripts induced in the lumbar spinal segment on day 7 of neuropathic pain; the abundance levels of these genes were modulated by repeated intraperitoneal administration of minocycline (30 mg/kg; 16 h and 1 h before sciatic nerve damage and then twice daily for 7 days). **(C)** The percentage analysis of the alterations in the CCI-induced gene expression profile in the spinal cord.

The microarray method was used to study the effect of minocycline on the expression changes caused by injury to the sciatic nerve (Figure 2). The analysis involved three groups of animals: N – naïve, control animals; V-CCI – animals that received water for injection seven days after sciatic nerve ligation; and MC-CCI – animals that received repeated intraperitoneal administration of minocycline (30 mg/kg *i.p*.; 16 h and 1 h before nerve damage and then twice daily for seven days after CCI (Figure 2). The DNA microarray method (Illumina RatRef-12 V1) was used to analyze the changes in mRNA levels in the lumbar segment of the spinal cord. The results of the microarray analysis are presented in the form of a heat map. Statistical analysis (ANOVA) revealed that, out of 22,500 tested mRNA transcripts, 93 transcripts displayed changes in their relative abundance following CCI, at an ANOVA threshold value of p < 0.001. Of these, 54 transcripts were not affected by minocycline administration, while the levels of the other 39 transcripts were modulated following minocycline administration. The results are presented as two main gene transcription patterns: A and B (N *vs*. V-CCI *vs*. MC-CCI) (Figure 2A, B).

### Transcripts not modulated by minocycline administration

Statistical analysis (ANOVA) revealed that 58% (54 transcripts) of the 93 examined transcripts (*RGD1560961*, *Ltc4s*, *LOC308350*, *Btk*, *Ms4a6a*, *LOC681932*, *Ptprc*, *RGD1566043*, *Irf1*, *Angpt4*, *Psmb4*, *Ifi47*, *RGD1359108*, *Ccr5*, *Tifa*, *RGD1562655*, *Ifngr1*, *Cd14*, *Jak3*, *Lgals3bp*, *Hla*-*dma*, *RGD1564553*, *Fam105a*, *Irf5*, *Plek*, *Rac2*, *C4*-*2*, *St14*, *Myo1f*, *Adap2*, *Mefv*, *Apobec1*, *LOC497841*, *Pik3ap1*, *Lyl1*, *Lgmn*, *C1qa*, *C1qb*, *Clec4a3*, *Glipr1*, *Coro1a*, *C1qc*, *Plac8*, *Tspo*, *Laptm5*, *Arhgap9*, *Tbxas1*, *Fcgr2b*, *LOC498276*, *Ncf1*, *Tlr7*, *Hhex*, *Tlr2*, and *Sart2*) were characterized by an increase in abundance following CCI, and minocycline administrations did not affect these changes (Figure 2A i C -pattern A). These tests were conducted at an ANOVA statistical threshold of p < 0.001 (N *vs*. V-CCI *vs*. MC-CCI).

### Transcripts modulated by minocycline administration

Statistical analysis (ANOVA) revealed that 93 transcripts showed changes in their relative abundance following sciatic nerve ligation. Of these, the levels of 39 (42%) transcripts were modulated following repeated injections of minocycline, and these transcripts were described as having transcription pattern B (Figure 2B, C).

### Transcription pattern B

#### Transcription pattern B1 – 10.75% of 93 transcripts

Ten transcripts (*Kmo*, *Pcdha3*, *Clec7a*, *Lep*, *Apobec3b*, *Fam22f*, *Card11*, *Slc7a7*, *Cd40*, *and Etaa1*) were characterized by an increase in mRNA abundance after CCI, and minocycline administrations reduced the level of these changes.

#### Transcription pattern B2 – 7.53% of 93 transcripts

Seven transcripts (*Rwdd3*, *LOC364991*, *Gimap5*, *RGD1305604*, *Gpr110*, *LOC287028*, *and HeyI*) were characterized by a decrease in mRNA abundance following CCI, and minocycline administrations reduced the level of these changes.

#### Transcription pattern B3 – 8.60% of 93 transcripts

Eight transcripts (*RGD1559604*, *Ms4a6a*, *Btg2*, *LOC498606*, *Wdr45*, *Fn1*, *Abt1*, and *Pla1a*) were characterized by an increase in mRNA abundance following CCI, and minocycline administrations enhanced the level of these changes.

#### Transcription pattern B4 – 15.05% of 93 transcripts

Fourteen transcripts (*Ldhb*, *LOC501111*, *LOC688240*, *Ebp*, *LOC499941*, *Nkx2*-*2*, *Dhcr7*, *Cnp*, *Rtn4*, *Tnnc2*, *Slc22a8*, *LOC290549*, *Car4*, and *Idi1*) were characterized by high baseline mRNA levels, which decreased following CCI and continued to decrease as a result of minocycline administrations.

## qPCR validation of the mRNA transcript changes in the spinal cord and DRG following minocycline *i.p*. administration seven days after CCI

### Genes from the B1 transcription pattern

#### Genes similarly regulated by minocycline in the spinal cord and DRG

##### Cd40

**Figure 3:**
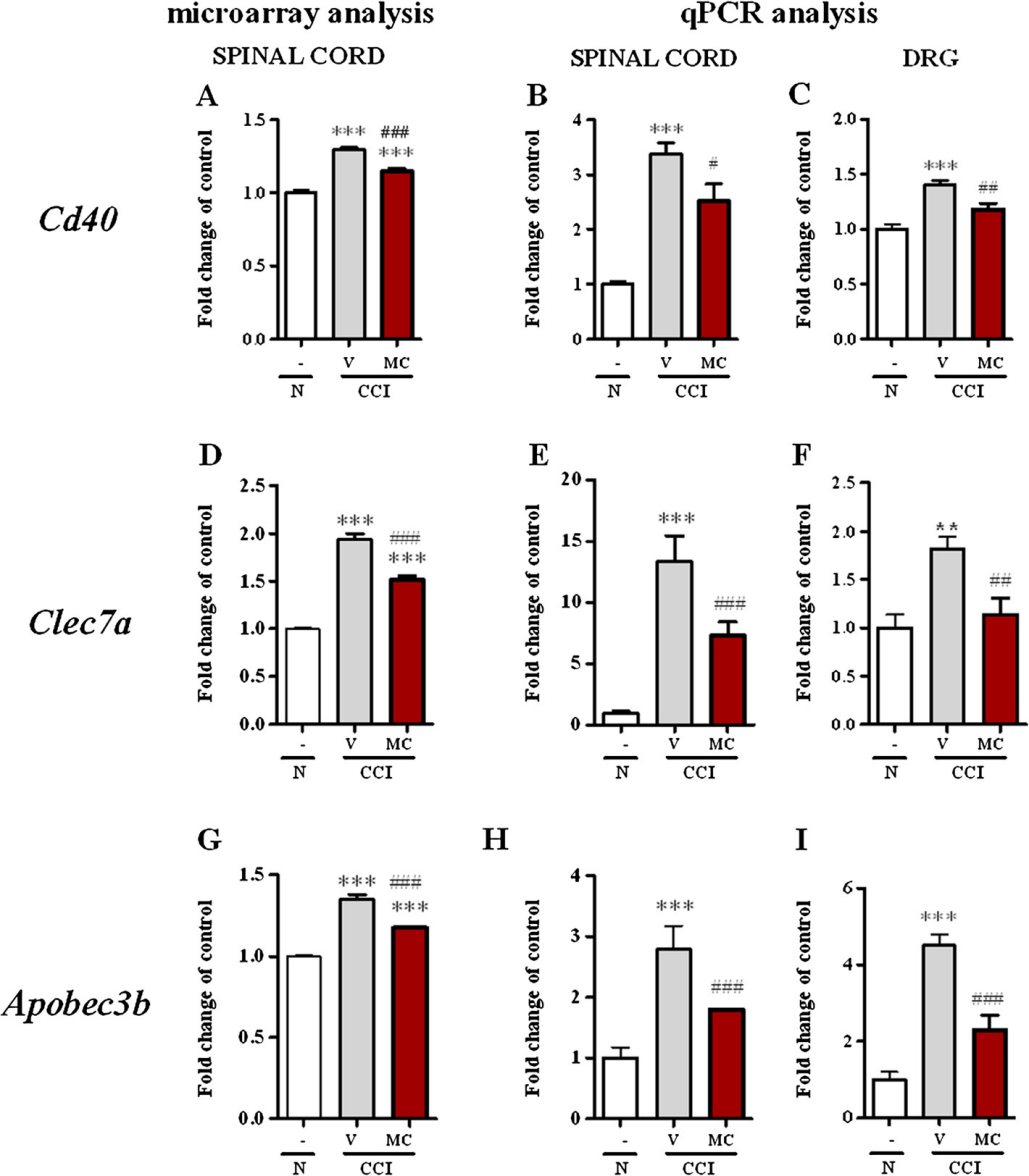
**Genes from the B1 transcription pattern** - **similarly spinally and DRG regulated by minocycline.** Comparison of the CCI-induced changes in transcript levels of genes with expression pattern B1 (*Cd40*, *Clec7a* and *Apobec3b*) in the lumbar spinal cord using microarray **(A, D, G)** and qPCR **(B, E, H)** analysis; transcript levels in the DRG **(C, F, I)** and the modulation by repeated intraperitoneal administration of minocycline (30 mg/kg; *i.p.* 16 h and 1 h before sciatic nerve damage and then twice daily for 7 days) were also examined. The statistical analysis was performed using an ANOVA and Bonferroni’s test. **p < 0.01 and ***p < 0.001 indicate a significant difference when compared to the control (naïve rats); ^#^p < 0.05, ^##^p < 0.01 and ^###^p < 0.001 indicate a significant difference when compared to the V-CCI rats. Naive (N), vehicle (V; water for injection), minocycline (MC).

In the dorsal lumbar spinal cord, we observed compared to naïve rats the upregulation of monocyte marker *Cd40* mRNA (1.0 ± 0.02 *vs*. 1.3 ± 0.009) using microarray analysis (Figure 3A). Microarray analysis of gene expression for T-cell (Cd3g, Cd3e, Cd3d, CD4, and CD8), B-cells (CD19) and NK-cells (CD335) markers suggest that there is no activation or infiltration of those cells into the spinal cord (Table 1). Minocycline significantly diminished the spinal level of *Cd40* mRNA (from 1.3 ± 0.009 to 1.1 ± 0.02).

**Table 1:** The influence of minocycline on lymphocyte and monocyte cell markers 7 days after CCI

**Cell Types**	**CDs**	**NAIVE**	**V-CCI**	**MC**-**CCI**
**Lymphocytes**				
T-cells	CD3 gamma	1.00 ± 0.03	0.90 ± 0.02	0.96 ± 0.02
T-cells	CD3 eppsilon	1.00 ± 0.06	0.92 ± 0.02	1.00 ± 0.01
T-cells	CD3 delta	1.00 ± 0.02	1.00 ± 0.01	1.09 ± 0.02
T-cells	CD4	1.00 ± 0.01	1.04 ± 0.01	1.15 ± 0,04
T-cells	CD8	1.00 ± 0.05	0.94 ± 0,01	1.03 ± 0.01
B-cells	CD19	1.00 ± 0.04	1.06 ± 0.02	0.96 ± 0.00
NK -cells	CD335	1.00 ± 0.04	1.06 ± 0.02	0.96 ± 0.00
**Monocytes**	**CD40**	**1.00** ± **0.02**	**1.3** ± 0.01^***^	**1.14** ± **0.02**^###^

Microarray analysis of gene expression in neuropathic pain and its modulation by repeated intraperitoneal administration of minocycline (30 mg/kg; 16 h and 1 h before sciatic nerve damage and then twice daily for 7 days) in the lumbar spinal cord. The results of the cDNA microarray analysis are presented as the mean ± SEM. The statistical analysis was performed using an ANOVA and Bonferroni’s test. ***p <0.001 indicates a significant difference when compared to the control (naive rats); ^###^p<0.001 indicates a significant difference when compared to the V-CCI rats. Naive (N), vehicle (V; water for injection), minocycline (MC).

qPCR analysis (Figure 3B) confirmed the changes detected by microarray. In the spinal cord, the upregulation of *Cd40* mRNA (1.0 ± 0.05 *vs*. 3.4 ± 0.2) was observed compared to the naïve rats. Minocycline significantly diminished the spinal level of *Cd40* mRNA (from 3.4 ± 0.2 to 2.5 ± 0.3). In the DRG (Figure 3C), the upregulation of *Cd40* mRNA (1.0 ± 0.04 *vs*. 1.4 ± 0.04) was observed compared to the naïve rats and minocycline significantly diminished the level of *Cd40* (from 1.4 ± 0.04 to 1.17 ± 0.05).

##### Clec7a

Using microarray analysis, in the dorsal lumbar spinal cord, we observed compared to naïve rats the upregulation of *Clec7a* mRNA (1.0 ± 0.009 *vs*. 1.9 ± 0.06) (Figure 3D) and minocycline significantly diminished the level of *Clec7a* mRNA (from 1.9 ± 0.06 to 1.5 ± 0.04).

qPCR analysis (Figure 3E) confirmed the changes detected by microarray. In the spinal cord, the upregulation of *Clec7a* mRNA (1.0 ± 0.009 *vs*. 13.4 ± 2.00) was observed compared to the naïve rats, and treatment with minocycline significantly diminished the level of *Clec7a* mRNA (from 13.4 ± 2.00 to 7.2 ± 1.1). In the DRG (Figure 3F), the upregulation of *Clec7a* mRNA (1.0 ± 0.13 *vs*. 1.8 ± 0.12) was observed compared to the naïve rats, and repeated treatment with minocycline significantly diminished the level of *Clec7a* mRNA (from 1.8 ± 0.12 to 1.14 ± 0.17).

##### Apobec3b

In the dorsal lumbar spinal cord, we observed compared to naïve rats the upregulation of *Apobec3b* mRNA (1.0 ± 0.008 *vs*. 1.3 ± 0.03) using microarray analysis (Figure 3G) and repeated treatment with minocycline significantly diminished the level of *Apobec3b* mRNA (from 1.35 ± 0.03 to 1.17 ± 0.007).

qPCR analysis (Figure 3H) confirmed the changes detected by microarray. In the spinal cord, the upregulation of *Apobec3b* mRNA (1.0 ± 0.2 *vs*. 2.8 ± 0.4) was observed compared to the naïve rats, and repeated treatment with minocycline significantly diminished the level of *Apobec3b* mRNA (from 2.8 ± 0.4 to 1.8 ± 0.002). In the DRG (Figure 3I), *Apobec3b* mRNA (1.0 ± 0.2 *vs*. 4.5 ± 0.3) was upregulated compared to the naïve rats, and repeated treatment with minocycline significantly diminished the level of *Apobec3b* in the DRG (from 4.5 ± 0.3 to 2.3 ± 0.4).

#### Genes differentially regulated by minocycline in the spinal cord and DRG

##### Slc7a7

**Figure 4:**
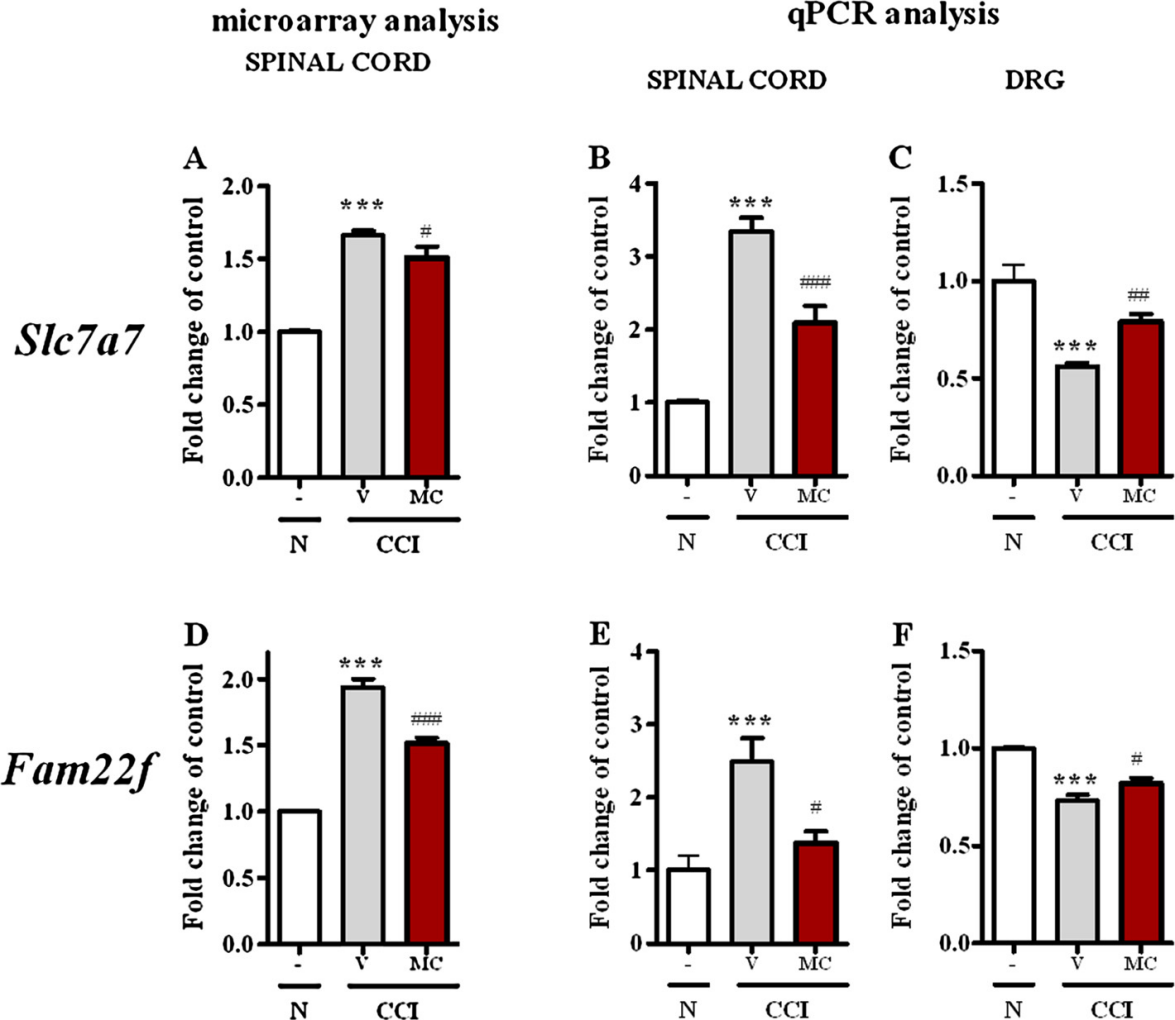
**Genes from the B1 transcription pattern** - **differentially spinally and DRG regulated by minocycline.** Comparison of the changes in the transcripts levels of genes with expression pattern B1 (*Slc7a7 and Fam22f*) in the lumbar spinal cord using microarray **(A, D)** and qPCR **(B, E)** analysis; transcript levels in the DRG **(C, F)** under neuropathic pain and the modulation by repeated intraperitoneal administration of minocycline (30 mg/kg; *i.p.* 16 h and 1 h before sciatic nerve damage and then twice daily for 7 days) were also examined using qPCR analysis. The statistical analysis was performed using an ANOVA and Bonferroni’s test. ***p < 0.001 indicates a significant difference when compared to the control (naïve rats); ^#^p < 0.05, ^##^p < 0.01 and ^###^p < 0.001 indicate a significant difference when compared to the V-CCI rats. Naive (N), vehicle (V; water for injection), minocycline (MC).

Using microarray analysis (Figure 4A), we observed compared to naïve rats the spinal upregulation of *Slc7a7* mRNA (1.0 ± 0.01 *vs*. 1.7 ± 0.03) and repeated treatment with minocycline diminished the level of *Slc7a7* mRNA (from 1.7 ± 0.03 to 1.5 ± 0.07).

qPCR analysis (Figure 4B) confirmed the changes detected by microarray, but only in the spinal cord samples. In the spinal cord, *Slc7a7* mRNA was upregulated (1.0 ± 0.04 *vs*. 3.3 ± 0.2) compared to the naïve rats and repeated treatment with minocycline significantly diminished the level of *Slc7a7* mRNA (from 3.3 ± 0.2 to 2.1 ± 0.2). In the DRG (Figure 4C), the downregulation of *Slc7a7* mRNA was observed (1.0 ± 0.1 *vs*. 0.6 ± 0.02), compared to naïve rats, and repeated treatment with minocycline increased the level of *Slc7a7* (from 0.6 ± 0.02 to 0.8 ± 0.04).

##### Fam22f

In the dorsal lumbar spinal cord, we observed compared to naïve rats the upregulation of *Fam22f* mRNA (1.0 ± 0.01 *vs*. 1.9 ± 0.06) using microarray analysis (Figure 4D) and repeated treatment with minocycline diminished the level of *Fam22f* mRNA (from 1.9 ± 0.06 to 1.5 ± 0.04).

qPCR analysis (Figure 4E) confirmed the changes detected by microarray, but only in the spinal cord. In the spinal cord, *Fam22f* mRNA (1.0 ± 0.02 *vs*. 2.5 ± 0.3) was upregulated compared to the naïve rats, and repeated treatment with minocycline significantly diminished the spinal level of *Fam22f* mRNA (from 2.4 ± 0.3 to 1.4 ± 0.2). In contrast, in the DRG (Figure 4F), the downregulation of *Fam22f* mRNA (1.0 ± 0.1 *vs*. 0.7 ± 0.03) was observed compared to the naïve rats, and repeated treatment with minocycline increased (from 0.7 ± 0.03 to 0.8 ± 0.03) the level of *Fam22f*.

### Genes from the B2 transcription pattern

#### Genes similarly regulated by minocycline in the spinal cord and DRG

##### Rwdd3

**Figure 5:**
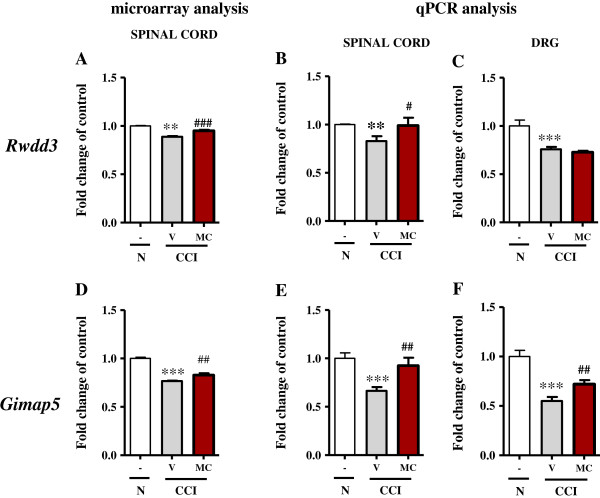
**Genes from the B2 transcription pattern** - **similarly spinally and DRG regulated by minocycline.** Comparison of the changes in the transcript levels of genes with expression pattern B2 (*Rwdd3* and *Gimap5*) in the lumbar spinal cord using microarray **(A, D)** and qPCR **(B, E)** analysis; transcript levels in the DRG **(C, F)** under neuropathic pain and the modulation by repeated intraperitoneal administration of minocycline (30 mg/kg; *i.p.* 16 h and 1 h before sciatic nerve damage and then twice daily for 7 days) were also examined using qPCR. The statistical analysis was performed using an ANOVA and Bonferroni’s test. **p <0.01 and ***p <0.001 indicate a significant difference when compared to the control (naïve rats); ^#^p < 0.05, ^##^p < 0.01 and ^###^p < 0.001 indicate a significant difference when compared to the V-CCI rats. Naive (N), vehicle (V; water for injection), minocycline (MC).

In the dorsal lumbar spinal cord, we observed compared to naïve rats a slight downregulation of *Rwdd3* mRNA (1.0 ± 0.004 *vs*. 0.8 ± 0.006) using microarray analysis (Figure 5A), and repeated treatment with minocycline resulted in an increase (from 0.8 ± 0.006 to 0.9 ± 0.008) in the level of *Rwdd3* mRNA.

qPCR analysis (Figure 5B) confirmed the changes detected by microarray. In the spinal cord, *Rwdd3* mRNA (1.0 ± 0.006 *vs*. 0.8 ± 0.05) was downregulated compared to the naïve rats, and repeated treatment with minocycline significantly increased the level of *Rwdd3* mRNA (from 0.8 ± 0.05 to 1.0 ± 0.08). In the DRG (Figure 5C), *Rwdd3* mRNA (1.0 ± 0.006 *vs*. 0.75 ± 0.02) was downregulated compared to the naïve rats, and repeated treatment with minocycline did not influence the level of *Rwdd3* (0.75 ± 0.02 *vs*. 0.72 ± 0.01).

##### Gimap5

In the dorsal lumbar spinal cord, we observed compared to naïve rats the downregulation of *Gimap5* mRNA (1.0 ± 0.01 *vs*. 0.7 ± 0.003) using microarray analysis (Figure 5D). Chronic treatment with minocycline decreased (from 0.7 ± 0.003 to 0.8 ± 0.02) the level of *Gimap5* mRNA.

qPCR analysis (Figure 5E) confirmed the changes detected by microarray. In the spinal cord, the downregulation of *Gimap5* mRNA (1.0 ± 0.06 *vs*. 0.7 ± 0.04) was observed compared to the naïve rats, and repeated treatment with minocycline significantly increased the level of *Gimap5* mRNA (from 0.7 ± 0.04 to 0.9 ± 0.08). In the DRG (Figure 5F), *Gimap5* mRNA was downregulated (1.0 ± 0.06 *vs*. 0.5 ± 0.04) compared to the naïve rats, and treatment with minocycline increased (from 0.5 ± 0.04 to 0.7 ± 0.04) the level of *Gimap5*.

## Discussion

The widely accepted definition of neuropathic pain is “pain arising as a direct consequence of a lesion or disease affecting the somatosensory system” [32]. Neuropathic pain is very challenging to manage because of the heterogeneity of its etiologies, symptoms and underlying mechanisms, as well as our limited understanding of its development and progression [33]. Hypersensitivity to thermal and mechanical nociceptive stimuli in animals was observed on day 7 following sciatic nerve injury in our experiments, and this result is in agreement with our previous studies, as well as others [7,15,34-38]. Recently, accumulating evidence has suggested that glial cell activation and neuroinflammation are critical for the development and maintenance of persistent pain [1,4,5,14,39]. The results obtained after administration of minocycline in neuropathic pain models suggest that it has a therapeutic potential; however, it is well known that the its mechanism of action is not selective. In our previously published studies, we demonstrated an increase in the activation of monocytes on day 7 after sciatic nerve injury in the lumbar spinal cord and/or in the DRG [7,15]. Other studies revealed microglia activation on day 2 following sciatic nerve injury, with its highest activation being observed between days 7 and 10 [1,7,15,40,41]; therefore, we choose day 7 for our microarray analysis. Although it is evident that the main cause of the attenuation of neuropathy is the inhibition of microglial activation, minocycline also acts through many other targets. Our studies allowed us to isolate a series of genes whose expression profiles followed several patterns after CCI and minocycline treatment. As shown by our studies, 93 out of 22,500 studied transcripts undergo abundance level changes following sciatic nerve injury; of these, 54 transcripts were not affected by the repeated administration of minocycline, while strong modulation was observed in 39 transcripts. These 39 genes are potential targets for minocycline and could be interesting from a therapeutic viewpoint.

The results obtained from microarray analysis revealed that the abundance levels of 54 transcripts increased following sciatic nerve ligation, but these genes were not affected by the repeated administration of minocycline; these genes are shown in Figure 2A in the results section. The roles that many of these genes play in neuropathy are not known; therefore, these genes will be the subjects of our future research. In the present study, we validated the expression changes of seven of the most interesting genes from the 39 genes whose abundance levels increased after sciatic nerve injury and were modulated by repeated administration of minocycline using qPCR analysis of spinal cord samples, and we further studied the mRNA levels of these genes in DRG samples from the same experimental scheme. Furthermore, we selected two gene expression patterns to study in detail: B1 and B2. The B1 transcription pattern contained 10 transcripts; the mRNA abundance levels of these genes increased following injury, and minocycline administration reversed or inhibited the effect of the injury. The B2 transcription pattern contained 7 transcripts; the mRNA abundance levels of these genes decreased after sciatic nerve ligation, and minocycline administration reversed the effect of the injury. According to the literature, we selected the following genes for qPCR analysis: *Cd40*, *Clec7a*, *Apobec3b*, *Slc7a7*, and *Fam22f* from pattern B1 and *Rwdd3* and *Gimap5* from pattern B2.

### *Cd40*, *Clec7a* and *Apobec3b* are genes from the B1 transcription pattern that are upregulated by sciatic nerve injury in the spinal cord and DRG and are diminished by minocycline

CD40, a 48 kDa cell surface tumor necrosis factor (TNF) family receptor, has been shown to be upregulated in microglia upon activation in both *in vitro* and *in vivo* studies. CD40 is also expressed by a wide variety of cells, such as neurons, dendritic cells, microglia, B cells, macrophages, keratinocytes, endothelial cells, thymic epithelial cells, fibroblasts and various tumor cells [42]. It is known that CD40-mediated microglia activation contributes to disease progression in a variety of neuroinflammatory diseases, such as multiple sclerosis, Alzheimer’s disease, and cerebral ischemia, leading to the production of a wide array of cytokines, chemokines, matrix metalloproteinases and neurotoxins [43,44]. After CD40 is bound, numerous signaling pathways are activated, leading to changes in gene expression and function. The interaction between CD40 and CD154 appears to be critical for a productive immune response, the upregulation of various costimulatory molecules (ICAM-1, VCAM-1, E-selectin, LFA-3, B7.1, B7.2, and class II MHC) and the production of numerous cytokines/chemokines (IL-1, IL-6, IL-8, IL-10, IL-12, TNF-α, MIP-1α, and MCP-1). Thus, signaling through CD40 in macrophages/microglia induces a number of soluble mediators that have important functional roles in the CNS (central nervous system). Macrophages/microglia have been shown to express CD40 in patients with multiple sclerosis [45], and CD40-deficient mice fail to develop these diseases [46,47]. The strategies used to attenuate inflammatory responses within the CNS by inhibiting the activation of macrophages and microglia (by suppressing CD40 expression) may be beneficial for a growing number of neuroinflammatory diseases. Others have shown that TGF-β and IL-4 inhibit the IFN-γ-induced CD40 expression in microglia [48,49]. In 2012, Cao et al. [50] showed that CD40 plays an important role in leukocyte infiltration into the lumbar spinal cord after L5 spinal nerve transection. Studies using a CD40 neutralizing Ab suggest that CD40 is required early on in order to promote the maintenance of injury-induced mechanical hypersensitivity. These data are consistent with our data, which showed that intraperitoneal administration of minocycline inhibited the development of mechanical allodynia and thermal hyperalgesia in parallel with the observed downregulation of CD40.

Clec7a, also called Dectin-1, was recently identified as the most important receptor for beta-glucan; Clec7a is a type II transmembrane protein that binds beta-1,3 and beta-1,6 glucans. Clec7a is primarily expressed by cells of myeloid origin, including monocytes, macrophages, microglia, neutrophils, most subsets of dendritic cells and a subset of T cells, B cells, mast cells, and eosinophils. Our studies using qPCR demonstrated that mRNA for *Clec7a* is expressed in rat primary microglial cells cultures (*date not shown*). Clec7a can recognize an unidentified endogenous ligand on T cells and may act as a costimulatory molecule; it can also induce a variety of cellular responses, including phagocytosis, respiratory burst and cytokine production [51]. Recently, Salazar-Aldrete et al. [52] showed that monocytes from patients with systemic lupus erythematosus and rheumatoid arthritis exhibited decreased expression of Clec7a in parallel with the enhanced synthesis of proinflammatory cytokines. Recent studies have highlighted the importance of Clec7a in anti-fungal immunity in both mice and humans and have suggested the possible involvement of this receptor in the control of mycobacterial infections [53]. Using microarray and qPCR analyses, we have shown that *Clec7a* mRNA is upregulated in the spinal cord and DRG at seven days after CCI-induced neuropathic pain and is downregulated after intraperitoneal administration of minocycline in the spinal cord and DRG.

Apobec3b is overexpressed in many tumors, including tumors found in the breast, cervix, bladder, lung, head and neck [54-57]. The overexpression of *Apobec3b* in animal models confirmed its tumor-type specificity [58]. It is not known which cells expressed *Apobec3b*, but after observing that minocycline downregulates the injury-increased level of *Apobec3b* mRNA, we hypothesize that microglia cells may be one type of cell that can express *Apobec3b*. Further studies are needed to clarify the role of this gene in the development of neuropathy. We observed a very similar pattern of *Apobec3b* transcription, as well as its regulation by minocycline, in the spinal cord and dorsal root ganglia, which suggest that microglia as well as other cell types, such as macrophages and leukocytes, can be the source of *Apobec3b*[58].

### Slc7a7 and Fam22f are genes from the B1 transcription pattern that are upregulated by sciatic nerve injury in the spinal cord, are downregulated in the DRG and are modulated by minocycline in both structures

Slc7a7 (*solute carrier family 7*, *amino acid transporter light chain*, *y* + *L system*, *member 7*) is an important amino acid transporter responsible for the sodium-independent influx/efflux of cationic and large neutral amino acids across the membrane [59]. Deregulation of the amino acid transporter Slc7a7 is involved in multiple types of cancer, including gliobastoma, non-small cell lung cancer and multiple myeloma [60-62]. Studies conducted in recent years have demonstrated that diseases associated with Slc7a7 also include lysinuric protein intolerance and cystinuria [63]. There are no reports detailing the roles Slc7a7 plays during neuropathic pain, inflammation or neurodegenerative disorders. Our study suggested that Slc7a7 may play a role after nerve injury. In the present study, using microarray and qPCR analyses, we showed that the level of *Slc7a7* mRNA is upregulated in the spinal cord seven days after CCI-induced neuropathic pain but is downregulated in the DRG. Repeated administration of minocycline reversed both of these changes. The regulation of this gene by minocycline in the spinal cord suggests it is present in microglia/macrophages. However, the involvement of the mononuclear phagocyte system appears to play a crucial role in the development of immunological complications, as was previously suggested by Barilli et al. [64]. The clinical significance of Slc7a7 expression in pain therapy needs to be clarified.

Fam22f’s role in neuropathic pain and/or neurodegenerative disorders needs to be described, as there is no information on this gene in the literature. Until now, it was only known that Fam22f belongs to the Fam22 family of transmembrane proteins, which span from one side of the membrane to the other side of the membrane. We tested this gene in the present study, and we demonstrated that *Fam22f* mRNA is upregulated in the spinal cord seven days after CCI-induced neuropathic pain but is downregulated in the DRG. Repeated administration of minocycline reversed both of these changes. The contrasting expression changes in the spinal cord and DRG suggest the participation of various cell types that express the gene in the tested structures after injury, but the role of minocycline in the expression of this gene remains to be explained.

### Rwdd3 and Gimap5 are genes from the B2 transcription pattern that are downregulated by sciatic nerve injury but are increased after minocycline treatment in the spinal cord and DRG

Rwdd3 belongs to the RWD domain family and was first described in 1996 by Bonaldo [65]. Recently, in 2013, Chi-Cheng Huang et al. [66] identified Rwdd3 as a gene associated with breast cancer risk. Some authors have suggested a role for Rwdd3 in neurosensory hearing loss and cellular stress [67]. Recently, Bergmann et al. [68] examined the association between the single nucleotide polymorphism rs2296308 in Rwdd3 and the development of neuropathy in paclitaxel-treated cancer patients, as was suggested by Schneider et al. [69]; however, this study was unable to confirm such a correlation. Our studies suggest the importance of Rwdd3 in the development of neuropathic pain, as we observed the CCI-induced upregulation of this gene, as well as its downregulation by chronic minocycline treatment.

Gimap5 refers to GTPase of immunity-associated nucleotide binding protein 5. Gimap5 is one of seven members of the Gimap family, which has been shown to be integral to T cell survival and development. These small GTPases regulate proapoptotic and antiapoptotic T cell pathways [70-73], as well as thymocyte maturation and differentiation [72,74]. There is a lack of information concerning the role or even regulation of Gimap5 during neuropathic pain; however, its important role in the function of the immune system suggests its importance. Our microarray and qPCR data strongly suggest it plays a significant role in the development of neuropathic pain. Our study using qPCR revealed the absence of *Gimap5* mRNA in rat primary microglial cell culture (*data not shown*). Until now, it has been known that a loss of Gimap5 function causes T cell lymphopenia in rats due to the near complete loss of post-thymic peripheral CD8 T cells, which triggers a lethal autoimmune disease [75]. It has also been shown that Gimap5 knockout mice lack peripheral NK cells and CD8+ T cells and exhibit dynamic changes in immune homeostasis, as demonstrated by the progressive loss of CD4+ T cells and B cells and the development of neutrophilia [76,77]. A frameshift mutation in Gimap5 is a prerequisite for the development of spontaneous type 1 diabetes in rats [72,73]. In 2011, Moralejo et al. [78] suggested that further elucidating the role of the Gimap5 in T cell survival, development and/or activation would aid in our understanding of the pathways involved in the onset of spontaneous diabetes mellitus type 1 and may ultimately uncover the pathways leading to development of this disease in humans. The mechanisms underlying the pro-survival function of Gimap5 in T cells have not been elucidated. Recently, some authors have shown that Gimap5-deficiency in T cells impairs Ca^2+^ entry via the plasma membrane channels [77]. Gimap5^sph/sph^ mice, an ENU germline mutant with a missense mutation in Gimap5, showed a progressive loss of the peripheral lymphocyte populations and developed spontaneous colitis, resulting in early mortality [79]. Genetic aberrations in Gimap5 have been linked to lymphopenia and the loss of immunological tolerance. Gimap5 is essential for maintaining lymphocyte quiescence and immunological tolerance. Its role during neuropathy needs further explanation and will be studied in our laboratory in the near future. In the present study, we have shown that *Gimap5* mRNA is downregulated in the spinal cord and DRG seven days after CCI-induced neuropathic pain. We also showed that intraperitoneal administration of minocycline inhibited the development of neuropathic pain symptoms and in parallel we observed that the level of *Gimap5* mRNA back to the level measured in naïve rats on the spinal cord level and DRG.

## Conclusions

To summarize, the DNA microarray method allowed us to choose 93 transcripts from 22,500 studied transcripts due to their abundance level changes after sciatic nerve injury, as well as 39 (42%) transcripts that were additionally modulated by repeated administration of minocycline. Further studies are needed to elucidate the roles of the individual genes in the development of neuropathic pain and to determine which genes altered by minocycline may be relevant to its analgesic action in neuropathic pain. The gene analysis in the present study offers the first step towards future research into genes that can be modulated and may be good targets for effective and safe therapies for the treatment of neuropathic pain.

## Materials and methods

### Animals

Male Wistar rats (300–350 g) from Charles River (Hamburg, Germany) were housed in cages lined with sawdust under a standard 12/12 h light/dark cycle (lights on at 08:00 h), with *ad libitum* access to food and water. All efforts were made to minimize animal suffering and to reduce the number of animals used in this study. All experiments were performed according to the recommendations of the International Association for the Study of Pain (IASP) [80] and the National Institutes of Health Guide for the Care and Use of Laboratory Animals, and these experiments were approved by the local Bioethics Committee (Krakow, Poland).

### Surgical preparations

Chronic constriction injury (CCI) was performed according to Bennett and Xie [81]. The right sciatic nerve was exposed under sodium pentobarbital anesthesia (60 mg/kg; *i.p*.). Four ligatures (4/0 silk) were made around the nerve, distal to the sciatic notch with 1 mm spacing, until a brief twitch in the respective hind limb was observed. After surgery, all rats developed symptoms of long-lasting neuropathic pain, such as allodynia and hyperalgesia.

### Drug administration and experimental scheme

**Scheme 1:**
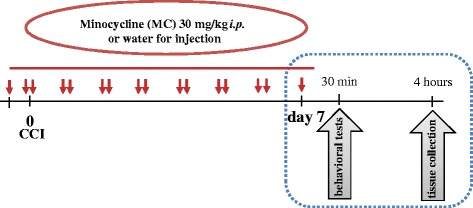
Drug administration and experimental schedule during 7 days after CCI. Drug administration and experimental schedule during 7 days after CCI.

Minocycline hydrochloride (30 mg/kg; Sigma, Schnelldorf, Germany) was dissolved in water for injection and administered preemptively by *i.p*. injection 16 and 1 h before CCI, then twice daily for seven days, as previously described [7,9]. This administration schedule was used because systemic microglia inhibitors attenuate the activation of microglia more efficiently when the inhibitor is injected before injury [9,27,82]. The control groups received the vehicle (water for injection) on the same schedule (Scheme 1).

According to the scheme, naive animals were subjected to all procedures (behavioral tests and tissue collection) parallel to the V-CCI and MC-CCI groups, with exception of CCI surgery procedure including anaesthesia. The influence of minocycline administration on gene expression in the ipsilateral dorsal part of the lumbar spinal cord (L4–L6) was studied using DNA microarray analysis. Injury-induced changes and the influence of minocycline were verified using the qPCR method, and the gene expression patterns in the ipsilateral DRG (L4–L6) were also examined.

### Behavioral tests

#### Tactile allodynia (von Frey test)

Allodynia was measured using an automatic von Frey apparatus (Dynamic Plantar Aesthesiometer Cat. No. 37400, Ugo Basile, Italy). The animals were placed in plastic cages with wire net floors 5 min before the experiment. The strengths of the von Frey stimuli used in our experiments ranged from 0.5 to 26 g. The von Frey test began with the lowest filament and increased in order; the filament was applied to the midplantar surface of the hind paw, and measurements were taken automatically, as described previously [7,15]. The ipsilateral paw was tested, and the mean value was calculated. There was almost no response to the highest strength (26 g) in the naive animals. Therefore, a line was drawn at this value. For the CCI-exposed rats, the significantly different reactions of the paws between the CCI-exposed and naive rats were recorded.

#### Cold hyperalgesia (cold plate test)

Hyperalgesia was assessed using the cold plate test (Cold/Hot Plate Analgesia Meter No. 05044, Columbus Instruments, USA) as previously described [7,15]. The temperature of the cold plate was kept at 5°C, and the cut-off latency was 30 s. The animals were placed on the cold plate, and the time until the hind paw was lifted was recorded. In the naive rat group, the reaction of the first hind paw to be lifted was measured. In the rats subjected to nerve injury, the ipsilateral paw reacted first.

### Biochemical tests

#### Tissue collection and RNA isolation

Ipsilateral and contralateral fragments of the dorsal part of the lumbar (L5–L6) spinal cord and the ipsi- and contralateral DRG (L5–L6) were removed immediately after decapitation on day 7 after CCI. The tissue samples were placed in individual tubes containing the tissue storage reagent RNAlater (Qiagen Inc.) and were stored at −70°C for RNA isolation. Total RNA was extracted using the TRIzol reagent (Invitrogen), as previously described [83]. The RNA concentration was measured using a NanoDrop ND-1000 Spectrometer (NanoDrop Technologies), and RNA quality was determined by chip-based capillary electrophoresis using an RNA 6000 Nano LabChip Kit and an Agilent Bioanalyzer 2100 (Agilent) according to the manufacturer’s instructions.

### Quantitative reverse transcriptase polymerase chain reaction (qPCR)

Reverse transcription was performed on 2 μg of total RNA using Omniscript reverse transcriptase (Qiagen Inc.) at 37°C for 60 min. RT reactions were carried out in the presence of an RNAse inhibitor (rRNAsin, Promega) and an oligo (dT16) primer (Qiagen Inc.). cDNA was diluted 1:10 with H_2_O, and for each reaction, ~ 50 ng of cDNA synthesized from the total RNA of an individual animal was used for the quantitative real-time PCR (qPCR) reaction. qPCR was performed using Assay-On-Demand TaqMan probes according to the manufacturer’s protocol (Applied Biosystems), and the reactions were run on an iCycler device (BioRad, Hercules). The following TaqMan primers and probes were used: Rn01527838_g1 (*Hprt*, hypoxanthine guanine rat hypoxanthine guanine phosphoribosyl transferase); Rn01522736_m1 (*Rwdd3*); Rn01772952_m1 (*Fam22f*); Rn00580189_m1 (*Slc7a7*); Rn01423590_m1 (*Cd40*); Rn00595553_m1 (*Gimap5*); Rn01505455_m1 (*Apobec3b*); and Rn01459401_m1 (*Clec7a*). The amplification efficiency for each assay (between 1.7 and 2) was determined by running a standard dilution curve. The cycle threshold values were calculated automatically by the iCycler IQ 3.0 software using the default parameters. RNA abundance was calculated as 2^-(threshold cycle)^. HPRT transcript levels do not significantly change in rats exposed to CCI [15] and, therefore, served as an adequate housekeeping gene.

### Microarray analysis

A starting amount of 200 ng of high quality total RNA was used to generate cDNA and cRNA using the Illumina TotalPrep RNA Amplification Kit (Illumina Inc., San Diego, CA, USA) according to the our previous studies [84,85]. The obtained cDNA served as a template for *in vitro* transcription with T7 RNA polymerase and biotin UTP to generate multiple copies of biotinylated cRNA. Each cRNA sample (1.5 μg) was hybridized overnight to a RatRef-12 V1 BeadChip array (Illumina); subsequently, the chips were washed, dried and scanned using the BeadArray Reader (Illumina). Raw microarray data were generated using BeadStudio v3.0 (Illumina). Samples from 2 rats were pooled for each microarray, and 3 biological replicates were used for each experimental point. Microarray quality control was performed using the BeadArray R package v1.10.0. The following parameters were checked: number of outliers, number of beads and percentage of detected probes. After background subtraction, the data were normalized using quantile normalization and were then log2-transformed. The obtained signal was taken as the measure of mRNA abundance derived from the level of its gene expression. All statistical analyses were performed using the R software version 2.11.1.

### Data analysis

The mean ± SEM of the behavioral data are presented in grams and seconds, and each group contained 6–15 rats. The results of the experiments were evaluated using one-way analysis of variance (ANOVA). The data are presented as the mean ± SEM. *p < 0.05, **p < 0.01 and ***p < 0.001 indicate a significant difference when compared to the control group (naïve rats); ^#^p < 0.05 and ^###^p < 0.001 indicate a significant difference when compared to the vehicle-treated CCI-exposed rats.

The results of the qPCR analyses are presented as the fold change compared to the control group (naive rats) and were calculated for the ipsilateral sides of the spinal cords, as well as for the DRGs of the CCI-exposed rats. The quantitative qPCR analysis data are presented as the mean ± SEM and represent the normalized averages derived from the threshold cycle in the qPCR analysis of 4 to 10 samples for each group. The inter-group differences were analyzed using an ANOVA, followed by Bonferroni’s multiple comparison test. *p < 0.05, **p < 0.01 and ***p < 0.001 indicate a significant difference when compared to the control group (naïve rats); ^#^p < 0.05 and ^###^p < 0.001 indicate a significant difference when compared to the vehicle-treated CCI-exposed rats.

Microarray analyses were performed in three groups: naïve, CCI-exposed and minocycline-treated CCI-exposed rats. The data are presented as fold changes compared to the naïve rats in the ipsilateral dorsal lumbar spinal cord and DRG. The inter-group differences were analyzed using ANOVAs, followed by the calculation of the false discovery rate (FDR). **p < 0.01 and ***p < 0.001 indicate significant differences when compared to the naïve rats. ^#^p < 0.05, ^##^p < 0.01 and ^###^p < 0.001 indicate significant differences when compared to the CCI-treated group.

## Abbreviations

CCI: Chronic constriction injury to the sciatic nerve; CNS: Central nervous system; DRG: Dorsal root ganglia; N: Naive; MC: Minocycline; IASP: International Association for the Study of Pain; V: vehicle.

## Competing interests

The authors declare that they have no competing interests.

## Authors’ contributions

ER - participated in the design of the study, behavioral and qPCR studies, analysis and interpretation of data, drafting of the manuscript. MK - microarray studies and analysis, data interpretation and discussion of results. RP - discussion of microarrays results, decision on the form of the manuscript BP - conception and design, develop and decision on the content of the manuscript JM - conception and design, supervised the experiments, drafting of the manuscript, elaboration and discussion of the whole manuscript. All authors have read and approved the final manuscript.
